# The POZ-ZF Transcription Factor Kaiso (ZBTB33) Induces Inflammation and Progenitor Cell Differentiation in the Murine Intestine

**DOI:** 10.1371/journal.pone.0074160

**Published:** 2013-09-05

**Authors:** Roopali Chaudhary, Christina C. Pierre, Kyster Nanan, Daria Wojtal, Simona Morone, Christopher Pinelli, Geoffrey A. Wood, Sylvie Robine, Juliet M. Daniel

**Affiliations:** 1 Department of Biology, McMaster University, Hamilton, Ontario, Canada; 2 Department of Pathology & Molecular Medicine, Queen’s University, Kingston, Ontario, Canada; 3 Department of Medical Sciences, University of Torino, Torino, Italy; 4 Department of Pathobiology, University of Guelph, Guelph, Ontario, Canada; 5 Department of Morphogenesis and Intracellular Signalling, Institut Curie-CNRS, Paris, France; Université Paris-Diderot, France

## Abstract

Since its discovery, several studies have implicated the POZ-ZF protein Kaiso in both developmental and tumorigenic processes. However, most of the information regarding Kaiso’s function to date has been gleaned from studies in *Xenopus laevis* embryos and mammalian cultured cells. To examine Kaiso’s role in a relevant, mammalian organ-specific context, we generated and characterized a Kaiso transgenic mouse expressing a murine Kaiso transgene under the control of the intestine-specific *villin* promoter. Kaiso transgenic mice were viable and fertile but pathological examination of the small intestine revealed distinct morphological changes. Kaiso transgenics (*Kaiso^Tg/+^*) exhibited a crypt expansion phenotype that was accompanied by increased differentiation of epithelial progenitor cells into secretory cell lineages; this was evidenced by increased cell populations expressing Goblet, Paneth and enteroendocrine markers. Paradoxically however, enhanced differentiation in *Kaiso^Tg/+^* was accompanied by reduced proliferation, a phenotype reminiscent of Notch inhibition. Indeed, expression of the Notch signalling target HES-1 was decreased in *Kaiso^Tg/+^* animals. Finally, our Kaiso transgenics exhibited several hallmarks of inflammation, including increased neutrophil infiltration and activation, villi fusion and crypt hyperplasia. Interestingly, the Kaiso binding partner and emerging anti-inflammatory mediator p120^ctn^ is recruited to the nucleus in *Kaiso^Tg/+^* mice intestinal cells suggesting that Kaiso may elicit inflammation by antagonizing p120^ctn^ function.

## Introduction

Since its discovery as a binding partner for the Src kinase substrate and cell adhesion protein p120^ctn^, mounting evidence suggests that the POZ-ZF transcription factor Kaiso functions in vertebrate development and tumorigenesis [1], [2], [3], [4], [5], [6], [7], [8]. To date however, Kaiso’s role in these processes in mammalian systems remains unclear, and much controversy surrounds several aspects of Kaiso’s function; this includes the mechanism by which it binds DNA [9], [10], [11], [12], [13], [14], [15], [16], [17] and its function in regulating the canonical Wnt signalling pathway that plays a key role in vertebrate development and tumorigenesis [8], [11], [14], [18], [19].

One study investigated the effect of Kaiso depletion on murine development and found that Kaiso null mice exhibited no overt developmental phenotypes [8]. This unexpected lack of a developmental phenotype may be attributed to the existence of two Kaiso-like proteins in mammals, ZBTB4 and ZBTB38, that may function redundantly with Kaiso [16], [20], and highlights what may be an important consideration in deciphering Kaiso’s role in mammalian systems. Surprisingly however, Kaiso depletion extended the lifespan, and delayed tumour onset in the *Apc^Min/+^* model of intestinal tumorigenesis [8]. This observation implicated Kaiso as an oncogene and is consistent with the report that Kaiso binds and represses methylated tumour suppressor and DNA repair genes in colon cancer cells [7]. Given that constitutive Wnt signalling resulting from mutation of *APC* functions as the first “hit” in *Apc^Min^* ^/+^-mediated tumorigenesis, the *Kaiso*-null/*Apc^Min^* ^/+^ phenotype suggests that Kaiso is a positive regulator of Wnt signalling. This result is surprising, since Kaiso has been implicated as a negative regulator of canonical Wnt signalling in *Xenopus laevis* embryos and in mammalian cultured cells [19], [21], [22], [23]. However it remains possible that Kaiso may potentiate intestinal tumorigenesis in the *Apc^Min^* ^/+^ model via a non-Wnt related mechanism.

Consistent with this possibility, studies to elucidate the role of the Kaiso binding partner p120^ctn^ in the intestine hinted at a non-cell autonomous mechanism for p120^ctn^-mediated tumorigenesis [24], [25]. Smalley Freed *et al.* found that mice with limited ablation of p120^ctn^ developed adenomas in addition to an intestinal barrier defect and chronic inflammation [25]. Surprisingly, conditional depletion of p120^ctn^ in the murine intestine resulted in severe **i**nflammatory **b**owel **d**isease (IBD) and lethality [24], [25]. Thus it was postulated that the adenomas arising in mice with limited p120^ctn^ ablation was a result of chronic inflammation, which is considered a risk factor for colorectal cancer [26].

Since studies have implicated Kaiso in intestinal cancer development and progression [7], [8], we generated an intestinal-specific Kaiso overexpression mouse model to clarify Kaiso’s role in the context of murine intestinal epithelium development. We generated multiple Kaiso transgenic (*Kaiso^Tg/+^*) founder lines, each with varying copy numbers of the transgene. *Kaiso^Tg/+^* mice were viable and fertile with no deleterious developmental phenotypes. However we noticed several phenotypes in the intestines of *Kaiso^Tg/+^* mice that were reminiscent of Notch inhibition. *Kaiso^Tg/+^* mice exhibited increased differentiation of intestinal epithelial progenitor cells into secretory cell lineages (Paneth, Goblet, enteroendocrine) accompanied by reduced proliferation, a phenotype consistent with Notch inhibition [27], [28], [29]. Indeed, expression of the Notch signalling target HES-1 was also reduced in *Kaiso^Tg/+^* mice. Interestingly, p120^ctn^ localized mainly to the nucleus in the small intestine in *Kaiso^Tg^* ^/+^ mice, and this was accompanied by increased infiltration of inflammatory cells and myeloperoxidase activity (a surrogate marker for inflammation) suggesting that *Kaiso^Tg^* ^/+^ mice are more susceptible to inflammation. Together these data suggest that Kaiso functions in a pro-inflammatory role in the murine intestine by antagonizing the anti-inflammatory functions of p120^ctn^.

## Materials and Methods

### Ethics Statement

All mouse work was conducted according to the guidelines of the McMaster University Animal Research Ethics Board (AREB). Protocols for mouse husbandry, breeding, genotyping and euthanasia were approved by AREB under Animal Utilization Protocol (AUP) 10-05-32. Euthanasia was achieved via CO_2_ asphyxiation followed by cervical dislocation.

### Generation of Villin-Kaiso Transgenic Mice

Kaiso transgenic mice were created at the London Regional Transgenic Facility, University of Western Ontario. Myc-tagged murine *Kaiso* (*mKaiso-MT*) was cloned downstream of the murine 9 Kb intestinal-specific *villin* promoter fragment in the pBluescript II vector provided by Dr. Sylvie Robine (Institut Curie, Paris, France) [30]. The *villin-mKaiso-MT* fragment was excised from the plasmid by restriction enzyme digest with *SalI*. The isolated fragment was microinjected into 1-cell C57BL6/CBA hybrid mouse embryos *in vitro*, which were then implanted into pseudopregnant foster mothers to produce transgenic founders. Transgenic pups were identified by **p**olymerase **c**hain **r**eaction (PCR) analysis of DNA from tail biopsies using primer pairs corresponding to sequences in the Myc tag and murine Kaiso (forward *5′-ATC ATC AAA GCC GGG TGG GCA-3′* and reverse *5′-TTT TCT ACT CTC CAT TTC ATT CAA GTC CTC-3′*). The transgenic lines were backcrossed with C57BL/6N mice (Taconic) for a minimum of 8 generations to obtain stable transgenic offspring, which initially produced three transgenic founder lines, followed by an additional four transgenic founder lines. All transgenic offspring were genotyped by PCR using DNA obtained from ear snips upon weaning. Mice were fed a standard mouse chow diet and breeders were housed in the disease-free barrier facility, while post-genotyping pups were housed in a specific pathogen free (SPF) room with 12 h/12 h light/dark cycle in accordance with McMaster Central Animal Facility’s (CAF) Standard Operating Procedures (SOPs).

### Transgene Copy Number

Copy number standards were prepared by spiking wild-type tail DNA with specified amounts of purified transgenic DNA. PCR was performed using standard DNA and transgenic DNA from each founder line using the primers described above. The intensity of the band amplified in each of the transgenic animals was compared to that of the standards to estimate transgene copy number.

### Mouse Tissue Harvest

Mice were sacrificed via CO_2_ asphyxiation according to the McMaster CAF SOPs. Small and large intestines were immediately removed from the sacrificed animals and flushed with cold **p**hosphate-**b**uffered **s**aline (PBS) on ice. Tissues were either flash frozen in liquid nitrogen for long term storage or rolled into “Swiss rolls” for fixation in 10% neutral-buffered formalin for 48 hours, followed by 70% ethanol dehydration at room temperature. The small intestine was divided into four equal sections for formalin fixation. Fixed tissues were sent to McMaster Core Histology Research Services for paraffin-embedding and sectioning at 5 µm within one week of tissue harvest, and placed onto glass slides for **i**mmuno**h**isto**c**hemical (IHC) analysis as outlined below.

### Morphological Analysis

Crypt depth and villi length were evaluated using **h**aematoxylin and **e**osin (H&E) stained slides from both transgenic lines (n = 3 mice per genotype/founder line). Paneth cells were counted as eosin-filled cells at the base of the crypts. **P**eriodic **A**cid-**S**chiff (PAS) stain for Goblet cells was performed by the McMaster Core Histology Research Services according to standard protocols. All images were collected using the Aperio ScanScope system, and ImageScope software was used for all measurements. For each small intestine, 800 open crypts and 80 complete villi were assessed per mouse by two independent blind observers. Student’s T-test was used to compare any observed differences for statistical significance using GraphPad Prism.

### Immunohistochemistry

Tissue slides were incubated in xylenes at room temperature for 10 min (2 washes) to remove paraffin, followed by rehydration in an ethanol gradient. Tissue was permeabilized with **T**ris-**b**uffered **s**aline with 0.05% **T**ween-20 (TBS-T), and antigen retrieval was accomplished by boiling samples in 10 mM sodium citrate buffer (pH 6.0). Endogenous peroxidase activity was quenched with 3% hydrogen peroxide in TBS. Slides were incubated in 5% normal goat serum (NGS), 10% bovine serum albumin (BSA) in TBS-T with avidin blocking solution (Vector Laboratories) for 1 hour at room temperature. For Lysozyme staining (Pierce), antigen retrieval was performed by treating tissues with 200 µg/mL of Proteinase K (Roche) solution in 50 mM Tris, pH 7.4 for 5 minutes, and blocked in 10% NDS in PBS with avidin blocking solution for 1 hour at room temperature. The slides were then incubated with biotin blocking solution (Vector Laboratories) and primary antibodies: rabbit anti-Kaiso polyclonal (gift from Dr. Albert Reynolds) at 1∶1000 dilution, and mouse anti-c-Myc (Santa Cruz) at 1∶60, rabbit anti-Lysozyme (Peirce) 1∶50 at 4°C overnight. For rat anti-Ki67 (DAKO at 1∶20 dilution), mouse anti-Synaptophysin (DAKO at 1∶20 dilution), rabbit anti-HES-1 (Santa Cruz at 1∶75 dilution) and rabbit anti-Cyclin D1 (US Biological at a 1∶100 dilution) staining, antigen retrieval was accomplished by boiling samples at 95°C in Target Retrieval Solution Citrate pH 6.0 (DAKO). Slides were blocked in 5% **n**ormal **d**onkey **s**erum (NDS) in TBS-T for Ki67 and Cyclin D1, in 5% NDS, 10% BSA in PBS for HES-1, and in 10% NGS, 10% BSA in PBS for Synaptophysin. Primary antibody incubation was performed for 2 hours at room temperature. After three 2-min washes in TBS-T, and one in TBS, slides were incubated in secondary antibodies (biotinylated donkey anti-rabbit [Vector Laboratories] at a 1∶1000 dilution, biotinylated goat anti-mouse [Vector Laboratories] at a 1∶1000 dilution, or biotinylated rabbit anti-rat [DAKO] at a 1∶200 dilution) for 2 hours at room temperature. Slides were washed as before, and incubated for 30 min in an avidin-biotin horseradish peroxidase complex, Elite ABC (Vector Laboratories). After a brief wash in TBS, Vectastain DAB substrate (Vector Laboratories) was applied for 3 minutes for satisfactory colour development. Ki67 and Cyclin D1 staining required a DAB time of 7 minutes. Tissues were counterstained with Harris hematoxylin (Sigma), differentiated in acid ethanol (0.3% HCl in 70% ethanol), blued in Scott’s tap water substitute, and dehydrated in a gradient of ethanol. Slides were then dried in xylenes and mounted using PolyMount (Polysciences Inc). Images were acquired using the Aperio ScanScope, and processed using ImageScope.

### Immunofluorescence

Tissue slides were incubated in xylenes at room temperature for 10 min (2 washes) to remove paraffin, followed by rehydration in an ethanol gradient as described above. Tissue was permeabilized with 0.05% TBS-T, and antigen retrieval was accomplished by boiling samples in 10 mM sodium citrate buffer (pH 6.0). Tissues were incubated in 5% normal goat serum, 10% bovine serum albumin in TBS-T for 1 hour at room temperature. The slides were then incubated with mouse monoclonal anti-p120 (BD Biosciences) at a dilution of 1∶500 at 4°C overnight. After three 10 min washes in TBS-T, and one in TBS, slides were incubated in secondary antibodies (Alexa-488 goat anti-mouse [Invitrogen], at a dilution of 1∶500) for 2 hours in the dark at room temperature. Slides were washed as before, and incubated for 30 min in the dark with TOTO-3 dye (Invitrogen; 1∶1000) to stain the nuclei. Slides were mounted in ProLong Gold (Invitrogen) overnight in the dark and stored at −20°C until imaging. Images were captured and processed using a Leica Confocal Microscope.

### Protein Isolation and Immunoblot

50 mg of flash frozen mouse tissue was minced with a sterile blade and homogenized in 1 mL cold RIPA buffer (1% NP-40, 50 mM Tris, 150 mM NaCl, 0.5% sodium deoxycholate, 1% SDS, 0.5% Na_3_VO_4_ and cOmplete ULTRA Tablet (1 tablet/5 mL buffer) [Roche]) in a chilled tissue grinder (Kontes). Harvested lysates were poured into chilled microfuge tubes followed by further homogenization using a 21 Gauge syringe. Lysates were incubated on ice for 30 minutes, followed by centrifugation at 13,000 RPM for 10 min at 4°C. The supernatants were transferred to new pre-chilled microfuge tubes. Total protein content was quantified by Bradford assay, and 25 µg of protein was resuspended in Laemmli sample buffer, boiled for 5 minutes and subjected to electrophoresis in an SDS polyacrylamide gel. Proteins were transferred to a nitrocellulose membrane using a Hoeffer semi-dry transfer apparatus (Amersham Biosciences). To prevent non-specific antibody binding, the membranes were blocked with 3% skimmed milk/TBS (pH 7.4) and incubated at 4°C overnight with antibody diluted in 3% milk/TBS. Antibodies used were as follows: anti-Kaiso rabbit polyclonal antibody at a 1∶30,000 dilution, anti-Cyclin D1 rabbit polyclonal antibody (US Biological) at a 1∶5,000 dilution, anti-β-actin mouse monoclonal antibody (Sigma Aldrich) at a 1∶30,000 dilution. The membranes were washed 5×5 minutes each with TBS and incubated at room temperature with HRP-conjugated donkey anti-mouse or goat anti-rabbit secondary antibody both at a dilution of 1∶40,000 in 3% milk/TBS. Membranes were washed as previously described and processed with Enhanced Chemiluminescence (Amersham Biosciences) according to the manufacturer’s protocol.

### RNA Isolation

Mouse tissue was homogenized and total RNA purified using the RNeasy Kit (Qiagen). Briefly, ∼20 mg frozen tissues were chopped finely with a clean blade, resuspended in 600 µl Qiagen Buffer RLT, and homogenized on ice in a glass tissue grinder. Lysates were further homogenized using a 21 Gauge needle and syringe on ice. Total RNA was then purified from the homogenized lysate using the RNeasy kit according to manufacturer’s instructions.

### RT-PCR

**R**everse **t**ranscriptase PCR (RT-PCR) analysis was performed using SuperScriptII One-Step RT-PCR with Platinum Taq (Invitrogen). Briefly, 1 µg of RNA was DNaseI treated (Invitrogen) to remove any genomic DNA contamination. 100 ng total RNA was used for each reaction with primers specific to the *villin-mKaiso* transcript and transcription factor II D (TFIID) as a loading control. The primer pairs used were as follows*: villin-mKaiso*: forward *5′-CAA CTT CCT AAG ATC TCC CAG GT-3′* and reverse *5′-CAA GGA GTT CAG CAG ACT GG -3′*; *TFIID*: forward *5′-CCA CGG ACA ACT GCG TTG AT-3′* and reverse *5′-GGC TCA TAG CTA CTG AAC TG-3′*. The RT-PCR program included one round of cDNA synthesis at 50°C for 30 minutes, followed by denaturation at 95°C for 2 minutes. Twenty five cycles of DNA amplification was performed as follows: denaturation at 95° for 30 sec, annealing at 56°C for 30 sec, and extension at 72°C. Final extension occurred at 72°C for 10 mins.

### Quantitative RT-PCR

Total RNA was purified from ∼ 20 mg of small intestinal tissue as described above. 1 µg of RNA was DNaseI treated (Invitrogen) to remove any genomic DNA contamination, and cDNA synthesis was accomplished using the SuperScript III First-Strand Synthesis System (Invitrogen). RNA abundance was compared using PerfeCTa SYBR Green SuperMix Reaction Mixes (Quanta Biosciences). The standard curve method was used to calculate relative expression of HES1 and Kaiso following normalization to the housekeeping gene, GAPDH, and then normalizing to the non-Tg tissue level. Primer sequences used are as follows: *villin-mKaiso* as stated above; *mHES1*: forward *5′-AAA ATT CCT CCT CCC CGG TG-3′* and reverse *5′-TTT GGT TTG TCC GGT GTC G-3′*; and *mGAPDH: forward 5′–ATG ACC ACA GTC CAT GCC ATC–3′* and reverse *5′-CCT GCT TCA CCA CCT TCT TG-3′*. Student’s T-test was used to determine significance using GraphPad Prism.

### Myeloperoxidase (MPO) Assay

Approximately 50 mg of flash frozen ileum and colon were homogenized in 50 mg/mL of 0.5% HTAB buffer (0.5% hexadecyltrimethylammonium bromide in 50 mM phosphate buffer, pH 6.0) via sonication at 30 Hz for 4 minutes. Homogenates were cleared by centrifugation at 12,000 rpm for 15 minutes at 4°C. MPO Assay was carried by adding 200 µL of o-dianisidine dihydrochloride solution (16.8 mg/mL o-dianisidine dihydrochloride in 5 mM phosphate buffer, pH 6.0 with 50 µL of 1.2% H_2_O_2_) to 96-well plates. Samples (7 µL) were added to each well of the 96-well plate in triplicate, and absorbance measured at 450 nm every 30 sec (3 readings). The MPO activity was measured in units (U), where 1 U represents the amount of MPO needed to degrade 1 µmoL of H_2_O_2_/minute at 25°C, which gives an absorbance of 1.13×10^−2^ nm/min. MPO activity in each sample was determined as the change in absorbance [ΔA(t_2_-t_1_)]/Δmin]/(1.13×10^−2^). MPO activity/mg of tissue was calculated by dividing MPO U by 0.35 mg of tissue (7 µL homogenate×50 mg/mL buffer). Student’s T-test was used to compare any observed differences for statistical significance using GraphPad Prism.

## Results

### Generation of *villin*-*Kaiso* Transgenic Mice

**Figure 1:**
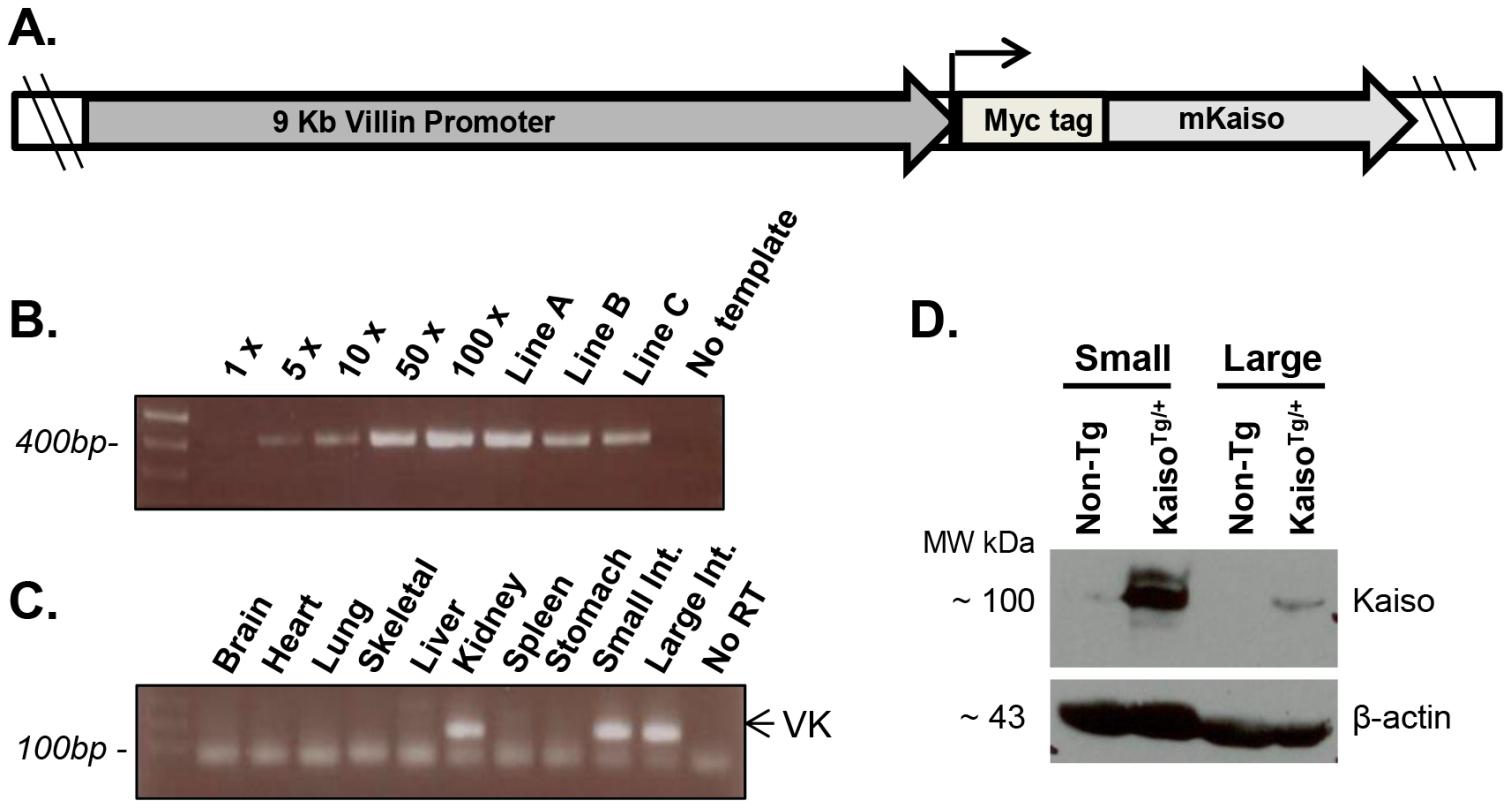
Generation of transgenic mouse lines ectopically expressing *villin*-Kaiso. (**A**) Myc-tagged murine *Kaiso* cDNA was cloned downstream of the 9 kb v*illin* promoter sequence. (**B**) The transgene copy number in each transgenic line was evaluated via PCR. Line A transgenic animals have the greatest copy number. (**C**) RT-PCR confirmed expression of the Kaiso transgene in *villin*-expressing tissues of transgenic mice, *i.e.* the small intestine, large intestine, and kidneys. (**D**) Immunoblot analysis shows increased Kaiso expression in both small and large intestines in Kaiso transgenic (*Kaiso^Tg^* ^/+^) Line A mice compared to non-transgenic (Non-Tg) siblings.

Kaiso transgenic (*Kaiso^Tg/+^*) mice were generated by cloning the sequence encoding N-terminal myc-tagged murine *Kaiso* downstream of a 9 Kb regulatory promoter region of the mouse *villin* gene (Figure 1A). The *villin-Kaiso* construct was injected into fertilized C57BL6/CBA embryos that were subsequently transferred to pseudopregnant foster mothers and resulted in four transgenic founder mice (Line A, B, C, D). Upon backcrossing with C57BL/6N mice, only lines A, B and C transmitted the transgene to their progeny at rates of 15%, 32% and 57%, respectively. Since pronuclear injections result in random genome integration, transgene copy number was estimated by PCR (Figure 1B). The three founders possessed varying copy numbers of the Kaiso transgene, with line A having the highest copy number and line C having the lowest copy number. Unfortunately, Line C died prior to being established and thus Lines A and B were used for further analysis. Upon founder line establishment (8 generations of backcrossing), Lines A and B transmitted the transgene at rates of 33.8% and 35.9% respectively, which is lower than the expected Mendelian rate of 50%.

To confirm tissue-specific expression of the *Kaiso* transgene, RT-PCR was performed with transgene-specific primers. As expected, the transgene was detected in all 3 villin-positive tissues: kidneys, small intestine and large intestine (Figure 1C). Kaiso protein expression was confirmed by Western blot analysis of protein harvested from small and large intestine (Figure 1D). Consistent with the transgene copy number observed via PCR, higher Kaiso protein expression was detected in Line A transgenics compared to Line B, with the lowest protein expression in Line C (data not shown).

**Figure 2:**
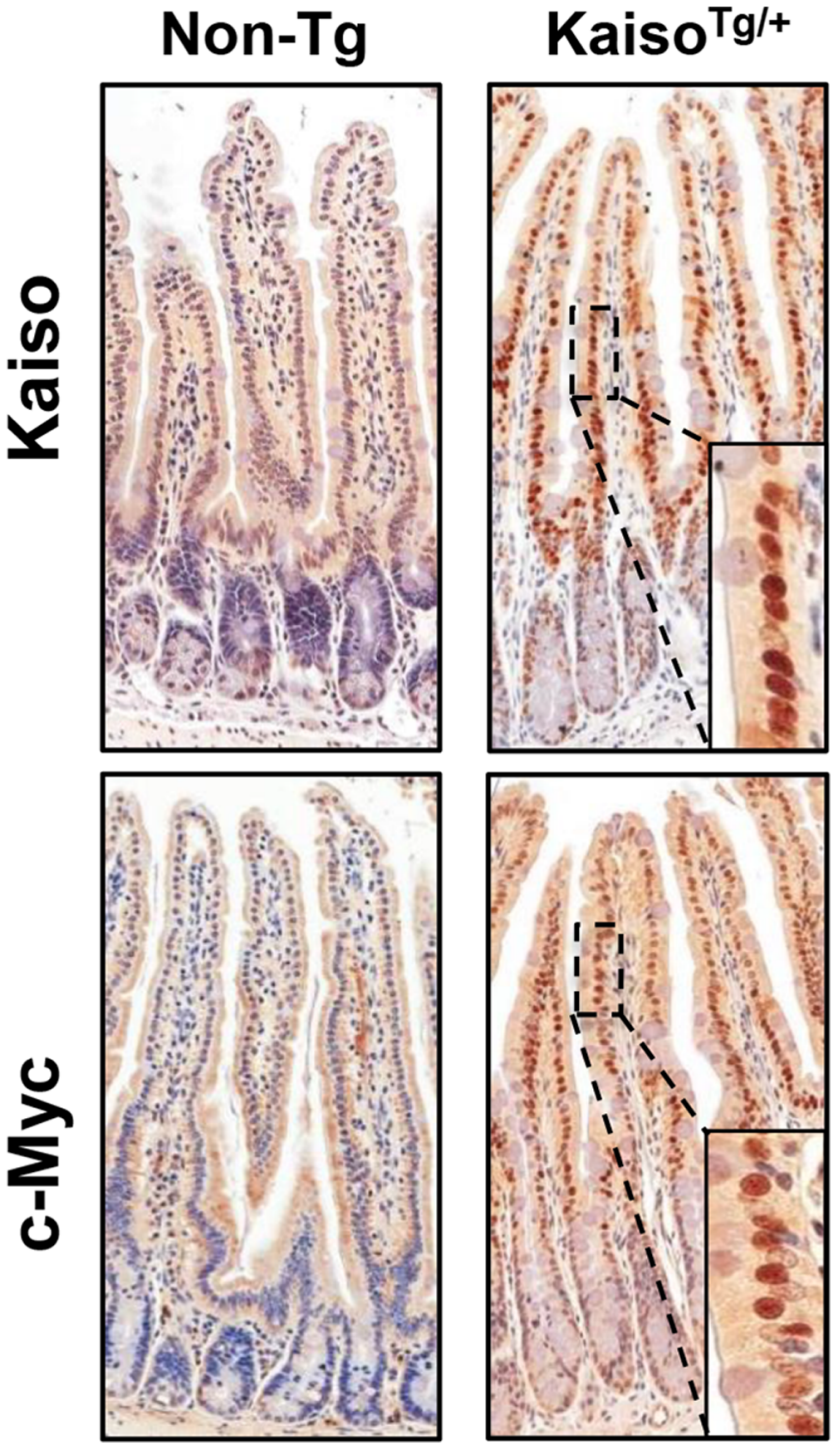
Subcellular localization and expression of ectopic Kaiso in Line A *Kaiso^Tg^* ^/+^ small intestines. *Kaiso^Tg^* ^/+^ mice display strong nuclear Kaiso in the villi and crypt cells, compared to non-transgenic mice (Non-Tg), which mainly display weak Kaiso staining in the cytoplasm. Additionally, *Kaiso^Tg/+^* mice display strong nuclear c-Myc staining corresponding to ectopic myc-tagged Kaiso expression, while Non-Tg mice display cytoplasmic c-Myc expression.

To further evaluate and confirm Kaiso expression and localization in *Kaiso^Tg/+^* and Non-Tg tissues, IHC was performed on tissues harvested from small and large intestines of Line A and Line B mice using a Kaiso-specific antibody. Line A *Kaiso^Tg/+^* mice exhibited stronger nuclear Kaiso expression in the villi and increased nuclear expression in the crypts of the small intestine compared to their Non-Tg siblings (Figure 2). However, Line B *Kaiso^Tg/+^*, which overexpressed less Kaiso than Line A, exhibited predominantly cytoplasmic localized Kaiso (Figure S1A). In the large intestine, both transgenic lines exhibited stronger Kaiso nuclear staining than their Non-Tg siblings (Figure S1B). Furthermore, strong nuclear Kaiso expression was observed in the epithelial cells near the top of the crypts, with lower expression at the bottom of the crypts (Figure 2). To confirm that increased Kaiso expression in *Kaiso^Tg/+^* mice was due to the transgene rather than an enhancement of endogenous *Kaiso* gene expression, we evaluated c-Myc expression in Line A small intestines. Indeed, *Kaiso^Tg/+^* mice exhibited stronger staining in comparison to Non-Tg mice, consistent with the expression of myc-tagged Kaiso (Figure 2). All subsequent analyses were performed on Line A *Kaiso^Tg/+^* (unless noted otherwise).

### Kaiso Transgenic Mice Exhibit Symptoms of Inflammation in the Intestinal Mucosa

**Figure 3:**
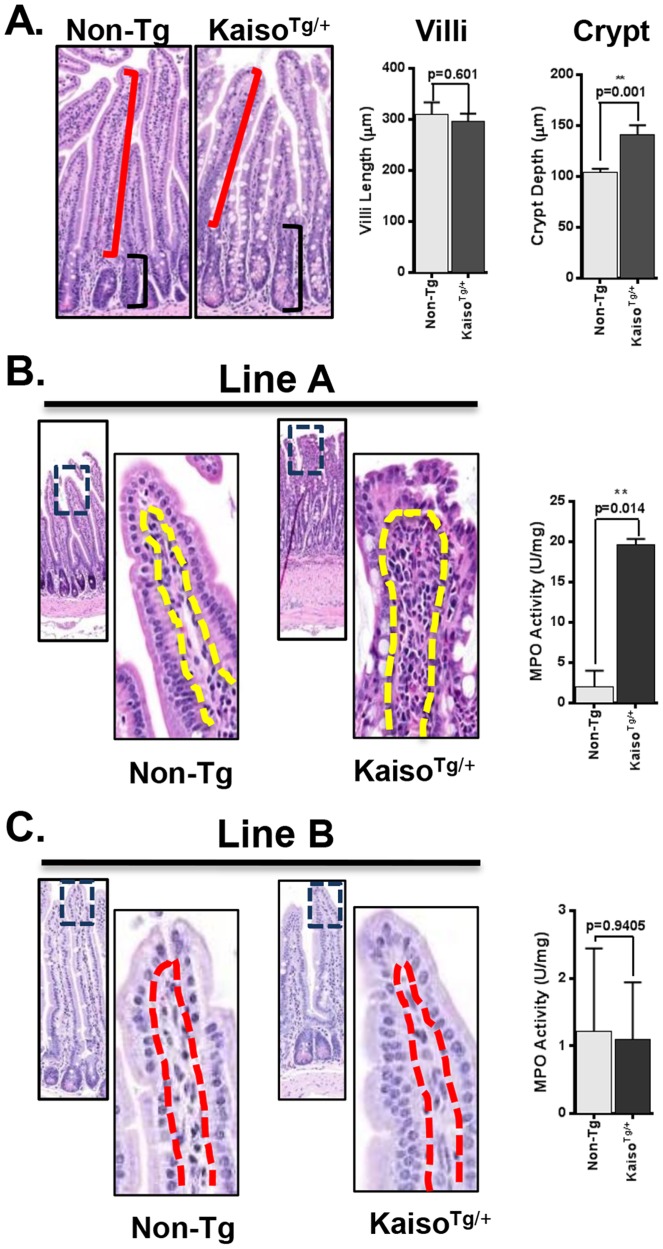
Kaiso transgenic mice exhibit inflammation of the intestinal mucosa. (**A**) **H**ematoxylin and **e**osin (H&E) stained sections were used to measure villi length (red bracket; ∼80 villi/mouse) and crypt depth (black bracket; ∼800 open crypts/mouse). *Kaiso^Tg^* ^/+^ display increased crypt depth compared to their Non-Tg siblings, p = 0.001. (**B**) *Kaiso^Tg^* ^/+^ mice exhibit increased immune cell infiltration of the lamina propria (yellow demarcated area) accompanied by increased MPO activity compared to their Non-Tg siblings, p = 0.014. (**C**) Line B mice do not exhibit immune cell infiltration or enhanced MPO activity compared to Non-Tg siblings. ** represents significance.

After establishing that Kaiso was robustly expressed in the intestine via our transgene we next sought to determine the effect of ectopic Kaiso on intestinal morphology and function. Examination of H&E stained sections from small and large intestinal tissues of 1-year old Line A mice revealed longer crypts with no difference in villi length in the small intestine (Figure 3A), although this phenotype was not observed in Line B mice. We also noticed that several villi were fused and blunted in our Line A *Kaiso^Tg/+^* mice in comparison to the characteristic elongated, finger-like appearance of villi in Non-Tg mice (Figure 3). To rule out the possibility that this phenotype was an artefact resulting from the transgene insertion site, we examined H&E sections from additional *Kaiso^Tg/+^* lines that had been backcrossed for only 3 generations (Lines D, E, F & G). Two of these lines, Lines E and F, exhibited even more robust Kaiso expression than Line A mice, concomitant with extensive villi fusion and blunting (Figure S2).

Crypt hyperplasia accompanied by fused, blunted villi has been previously reported in both humans and mice exhibiting chronic inflammation of the intestinal mucosa [31], [32], [33], [34], suggesting that ectopic Kaiso expression may cause intestinal inflammation. Indeed, closer examination of *Kaiso^Tg^* ^/+^ intestines (Line A, E and F) revealed increased immune cell infiltration of the lamina propria compared to their Non-Tg siblings (Figure 3B and Figure S2); however no such phenotype was observed in Line B mice with low ectopic Kaiso expression (Figure 3C). We also measured the levels of **m**yelo**p**er**o**xidase (MPO), which is a surrogate marker for inflammation, in *Kaiso^Tg/+^* and Non-Tg intestinal tissues. MPO activity was increased in the distal small intestine (ileum) of Lines A, E and F *Kaiso^Tg^* ^/+^ mice compared to their age-matched Non-Tg siblings (Figure 3B and Figure S2), while no change in MPO activity was detected in Line B mice (Figure 3C). Interestingly, the proximal colon of Lines E and F also exhibited increased MPO activity while mice from Lines A and B exhibited no such change (data not shown). These data suggest that ectopic Kaiso expression may predispose the murine intestine to inflammation, but this effect may be dose-dependent.

### Ectopic Kaiso Overexpression Results in Nuclear Accumulation of p120^ctn^

**Figure 4:**
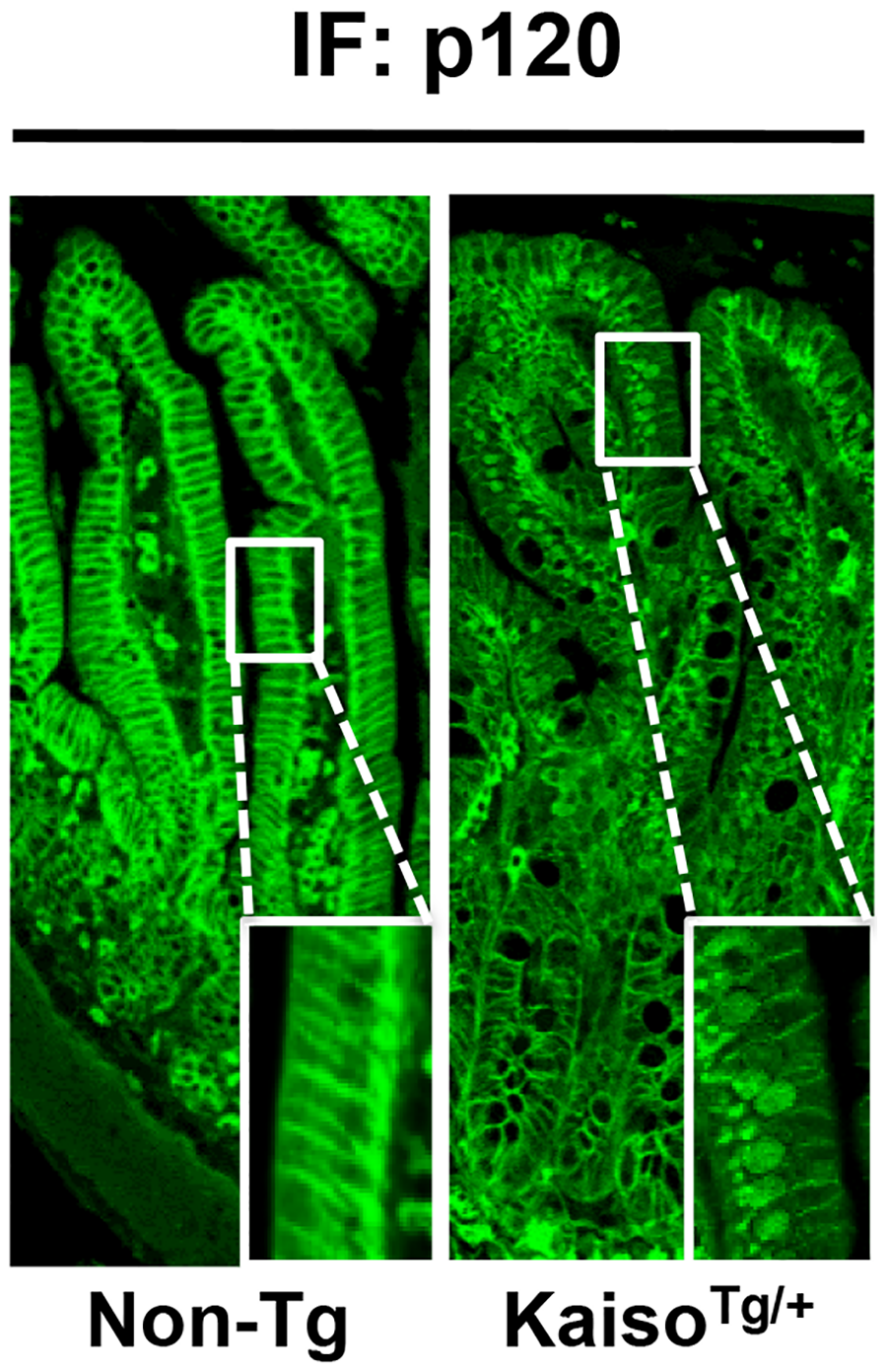
*Kaiso^Tg^* ^/+^ mice display nuclear p120^ctn^ in villi of the small intestine. Immunofluorescence staining for p120^ctn^ showed nuclear localization of p120^ctn^ in epithelial cells of villi overexpressing Kaiso (*Kaiso^Tg^* ^/+^), while Non-Tg mice displayed membrane localized p120^ctn^.

Given that *Kaiso^Tg/+^* mice exhibited an inflammatory response similar to that elicited by limited p120^ctn^ depletion [24], albeit less severe, we examined p120^ctn^ expression in the small intestines of our *Kaiso^Tg/+^* mice. Interestingly, in *Kaiso^Tg/+^* mice we observed nuclear localization of p120^ctn^ and reduced p120^ctn^ staining at the membrane in the distal small intestine (Figure 4). However in Non-Tg siblings, p120^ctn^ was largely membrane bound (Figure 4). Taken together this data suggests that Kaiso overexpression results in nuclear accumulation of p120^ctn^, and decreased membrane-bound p120^ctn^, which phenocopies the consequences of p120^ctn^ depletion [25].

### *Kaiso^Tg/+^* Mice Exhibit Enhanced Differentiation of Progenitor Cells into Secretory Cell Fates

**Figure 5:**
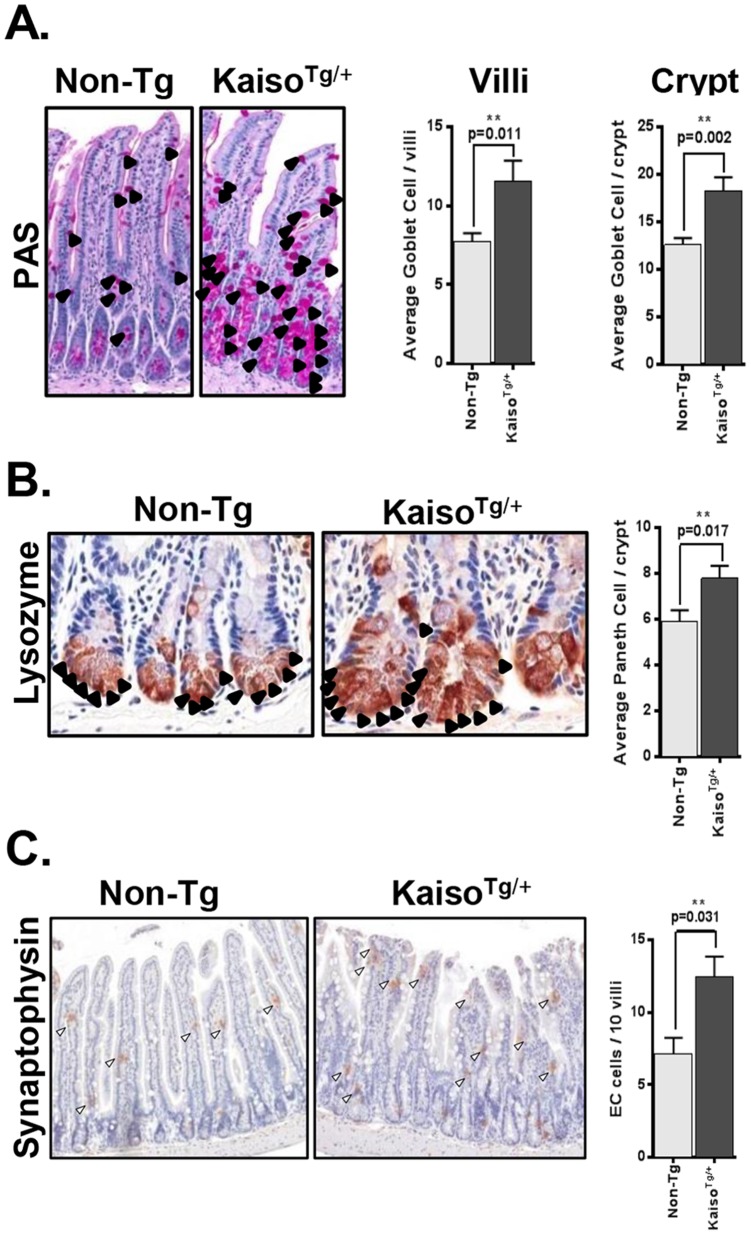
Secretory cell lineages are expanded in the intestines of *Kaiso^Tg^* ^/+^ mice. (**A**) PAS stain for Goblet cells (black arrowheads) revealed increased numbers of Goblet cells in both the villi and crypts of *Kaiso^Tg^* ^/+^ intestines, p = 0.011 & 0.002. (**B**) Lysozyme staining revealed increased Paneth cell numbers in *Kaiso^Tg^* ^/+^ mice, p = 0.017. (**C**) Synaptophysin positive enteroendocrine cells (arrowheads) are increased in *Kaiso^Tg^* ^/+^ mice, p = 0.031. n = 3 mice/genotype; measurements performed by two independent blind observers; T-test used for p-value. ** represents significance.

While characterizing the effect of ectopic Kaiso expression on intestinal morphology, we noted a significant expansion of Goblet cells in both the small and large intestine of Line A *Kaiso^Tg/+^* mice. Thus, we performed PAS staining for the Goblet cell-specific marker, Mucin, and quantification of Mucin positive (+) cells confirmed a significant increase in the Goblet cell population in both the small and large intestines of Line A mice compared to Line B and Non-Tg mice (Figure 5A & Figure S3). Interestingly, staining for the Paneth and enteroendocrine markers, lysozyme and synaptophysin respectively, revealed that these cell populations were also expanded in the small and large intestine of Line A *Kaiso^Tg/+^* mice but not in Line B or Non-Tg mice (Figure 5B, C & Figure S3).

**Figure 6:**
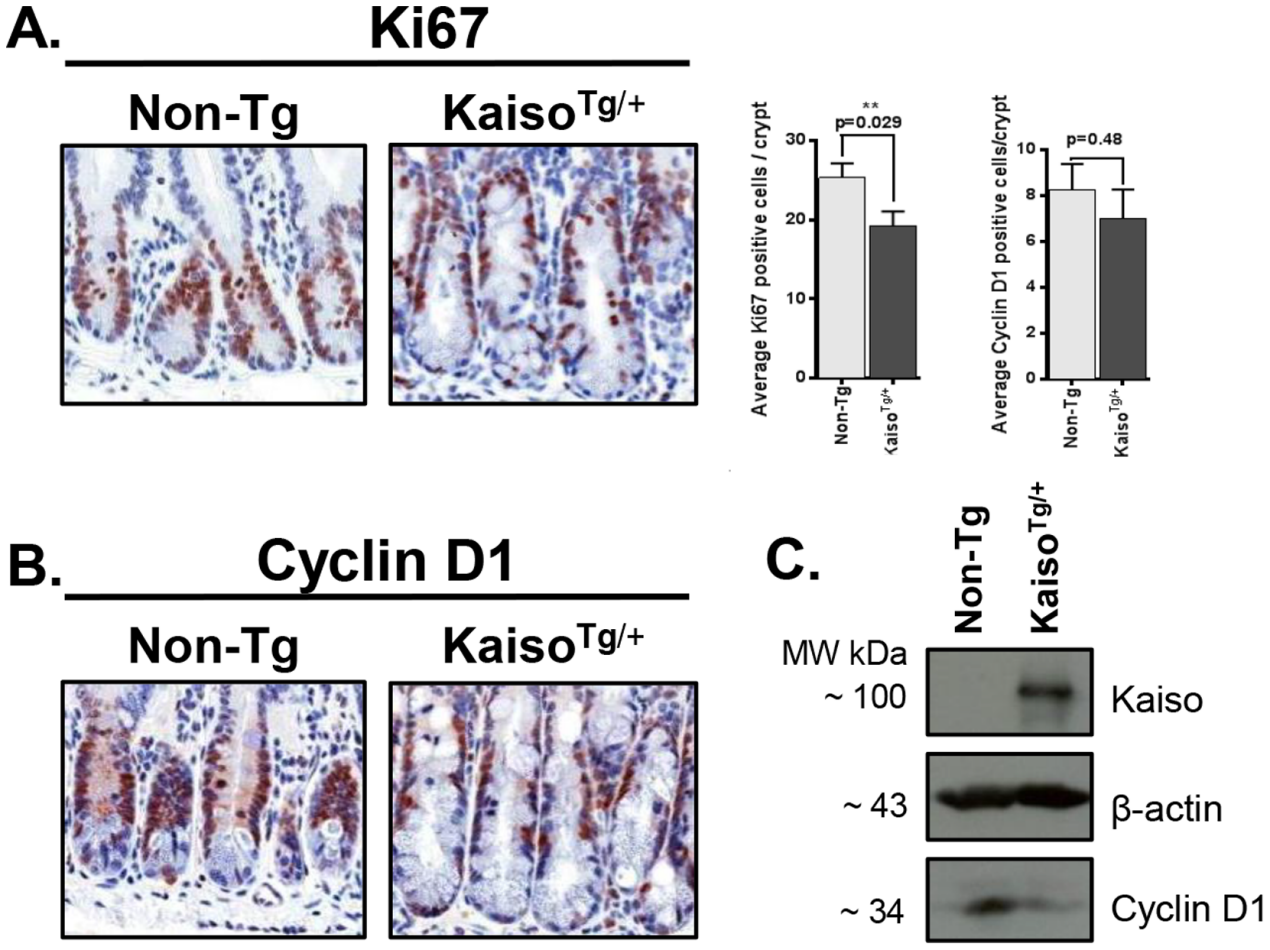
Cell proliferation is decreased in *Kaiso^Tg^* ^/+^ mice. Cell proliferation was evaluated by Ki67 (**A**) and Cyclin D1 (**B**) staining. Both markers exhibited reduced staining in *Kaiso^Tg^* ^/+^ mice compared to their Non-Tg siblings. Reduced CyclinD1 expression was also confirmed by immunoblot analysis of 3 different mice intestines (**C**). ** represents significance.

The expansion of secretory cell lineages in our *Kaiso^Tg/+^* mice led us to hypothesize that Kaiso may be driving progenitor cell differentiation. However, since we also observed crypt expansion in *Kaiso^Tg/+^* mice, we questioned whether the increase in secretory cells was indicative of increased progenitor cell proliferation. Hence we examined the expression of the cell proliferation marker Ki67. Surprisingly, Ki67 expression was decreased in Line A mice and Ki67 positive cells were localized more apical to the normal crypt/villus boundary (Figure 6A). We next evaluated the expression of the Kaiso target gene *cyclin D1* [4], [21] that has been shown to drive proliferation in the intestinal epithelium and is frequently overexpressed in colon cancer [35]. Similar to Ki67, Cyclin D1 expression was also decreased in Line A *Kaiso^Tg/+^* mice but surprisingly the apparent decreased numbers of Cyclin D1 positive (+) cells in *Kaiso^Tg/+^* intestines was not statistically significant (Figure 6B, C).

**Figure 7:**
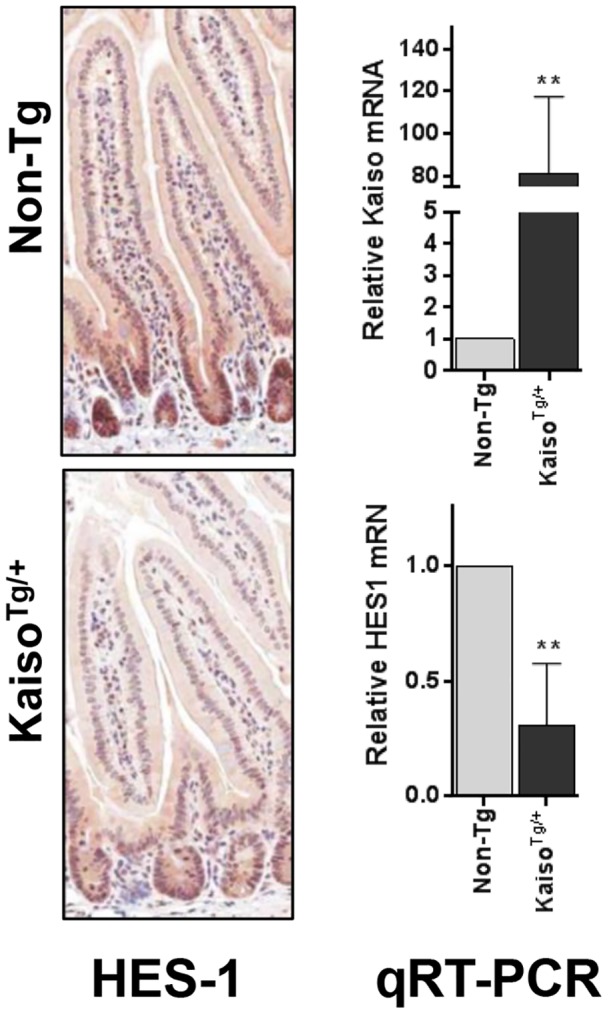
*Kaiso^Tg^* ^/+^ mice display decreased HES-1 expression in the small intestine. Both Non-Tg and *Kaiso^Tg^* ^/+^ tissues displayed nuclear HES-1 expression in the crypts of the small intestine, however *Kaiso^Tg^* ^/+^ tissue displays significantly decreased HES-1 expression in the villi. Quantitative RT-PCR showed a significant decrease in HES-1 expression in *Kaiso^Tg^* ^/+^ mice. Values were first normalized to the GAPDH housekeeping gene, followed by normalizing to non-Tg HES-1 expression (** represents p<0.05).

Previous studies have reported an expansion of secretory cell lineages and a reduction in the number of proliferating columnar base cells upon inhibition of the Notch signalling pathway in the intestine [28], [29], [36], [37]. Specifically, depletion of the Notch target gene *Hes-1* resulted in increased expression of secretory cell markers in the intestine of *Hes-1* null mice, suggesting that Hes-1 is necessary for specification of secretory cells in the intestine [37]. This prompted us to examine the expression of Hes-1 in our *Kaiso^Tg/+^* mice. Line A *Kaiso^Tg/+^* mice exhibited decreased Hes-1 staining and reduced expression of *Hes-1* mRNA compared to Non-Tg littermates (Figure 7). Together our data demonstrate that ectopic Kaiso elicits enhanced differentiation of progenitor cells into secretory lineages, perhaps through the down-regulation of the Notch target *Hes-1*.

## Discussion

Since Kaiso’s discovery over a decade ago, several studies have utilized *Xenopus laevis* and cultured cells as models to elucidate Kaiso’s biological roles [1], [2], [8], [15], [38], [39], [40], [41]. Here we describe the first study to examine the role of Kaiso in a relevant organ-specific context, the murine intestine. Using the murine *villin* promoter we were able to successfully drive intestinal-specific expression of the *Kaiso* transgene. In all founder lines, Kaiso was expressed along the entire crypt-villus axis with the most robust expression in the villi, which is consistent with the normal expression pattern of villin [30].

A previous report examining the effect of Kaiso depletion on *Apc^Min/+^*-mediated tumorigenesis found that Kaiso depletion resulted in fewer tumours [8], suggesting that Kaiso functions as an oncogene. However ectopic Kaiso expression was not sufficient to drive spontaneous tumour formation in our mouse model. Nonetheless, our Kaiso*^Tg/+^* Line A, mice exhibited enlarged crypts accompanied by fused, blunted villi, increased immune cell infiltration and increased MPO activity (indicative of neutrophil accumulation and inflammation) suggesting that *Kaiso^Tg^* ^/+^ mice have greater susceptibility to inflammation. Indeed, preliminary cytokine analysis of *Kaiso^Tg^* ^/+^ intestinal tissue revealed increased activity of the pro-inflammatory cytokine TNF-α compared to Non-Tg intestines (data not shown). Analysis of additional Kaiso transgenic lines (Lines E and F) revealed a similar intestinal phenotype to Line A, with concomitant increased neutrophil activation as measured by MPO activity. Increased MPO activity is often correlated with **u**lcerative **c**olitis (UC), a form of IBD and patients with IBD are at a higher risk of colon cancer [26], [42], [43], [44]. Thus in accordance with Knudson’s multiple hit theory of tumorigenesis, it is possible that Kaiso’s full oncogenic potential may only be unmasked in the presence of a second oncogenic insult such as *Apc* mutation or p53 loss of function. Intriguingly, preliminary analysis of intestinal tissues from a **d**extran **s**odium **s**ulfate (DSS)-induced model of colitis (kind gift of Dr. Elena Verdú), revealed increased expression of Kaiso compared to non-DSS treated mice (Figure S4), further supporting the notion that Kaiso overexpression plays a role in intestinal inflammation.

The enhanced inflammation observed in *Kaiso^Tg^* ^/+^ mice may be linked to altered p120^ctn^ function. Kaiso overexpression resulted in the nuclear localization of p120^ctn^, suggesting that Kaiso may somehow recruit or sequester p120^ctn^ to the nucleus. Given that p120^ctn^ was mainly localized to the cytoplasm and the cell membrane in non-transgenic mice, this change in localization may be indicative of altered or reduced p120^ctn^ function that may phenocopy p120^ctn^ loss observed by Smally Freed *et al.* [25]. Future studies are needed to determine whether p120^ctn^ directly contributes to the Kaiso overexpression phenotype.

Interestingly, the phenotypes observed in Lines A, E and F mice were not observed in Line B mice which express significantly lower levels of ectopic Kaiso; this suggests that a threshold level of Kaiso expression is necessary for the observed inflammatory phenotype. Additionally, no change in MPO activity was seen in Line B mice, further supporting our hypothesis of threshold effects of Kaiso expression. This is not surprising since varying amounts of Kaiso were shown to have completely opposite effects in *Xenopus laevis* embryos [18]. Hence in Line B mice, it is likely that Kaiso expression is below the threshold at which it elicits inflammation and leads to expanded crypts.

Finally, *Kaiso^Tg/+^* mice exhibited increased populations of Goblet, Paneth and enteroendocrine cells. This expansion of secretory cell populations accompanied by decreased cell proliferation is consistent with the phenotype observed upon pharmacological inhibition of Notch signalling [28] and in Hes-1^null^ mice [37]. One study found that Notch signalling is activated in intestinal epithelium in response to inflammation and is required for proper regeneration of the intestinal epithelium following colitis induced damage [45]. It should be noted that 90-day old *Kaiso^Tg/+^* mice exhibit increased Goblet cells but do not exhibit any overt signs of inflammation or myeloperoxidase activity (data not shown). This suggests that inflammation in these mice develops over time although Notch inhibition is present at a very early age. Thus it is possible that the intestinal epithelium in our *Kaiso^Tg/+^* mice is incapable of regeneration following bacterial or physical insult and consequently develops chronic inflammation over time.

**Figure 8:**
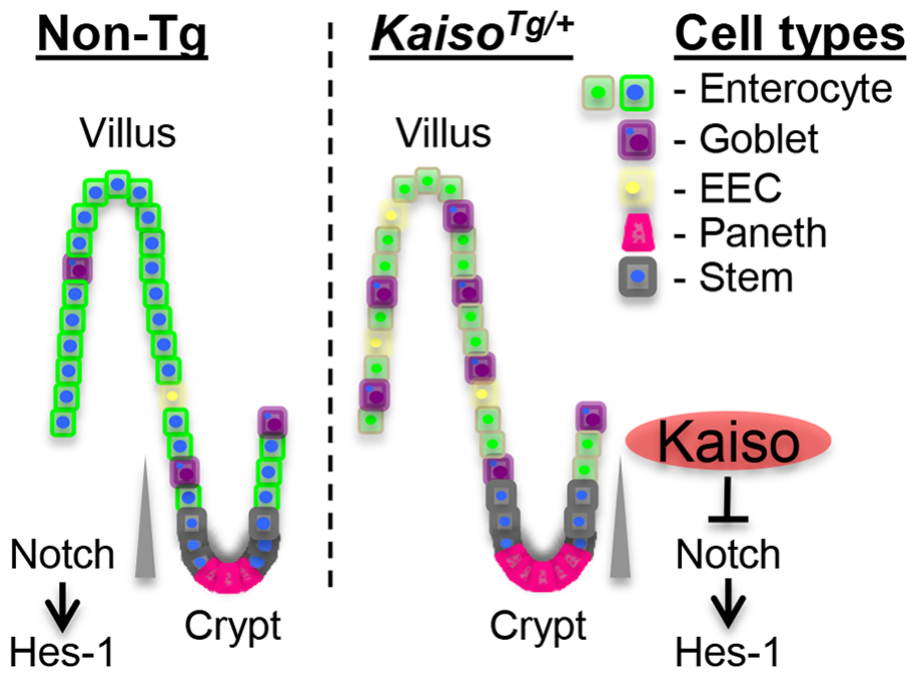
Schematic model of Kaiso’s postulated effects in the intestine. Notch signalling in the crypts modulates differentiation of progenitor cells into the various epithelial cell lineages: enterocytes, Goblet, Paneth and **e**ntero**e**ndo**c**rine (EEC) cells. The gradient of Notch signaling is indicated by the grey triangle. HES-1 is necessary for the proper specification of these cell types. p120^ctn^ localizes to the membrane in the enterocytes of Non-Tg mice (green-membraned cells), but is recruited to the nucleus in *Kaiso^Tg^* ^/+^ mice (green nucleated cells), which inhibits Notch signaling and Hes-1 expression, thus inducing inflammation.

In summary, Kaiso overexpression promotes inflammation and inhibits Notch signalling in the murine intestine. These findings support a model in which *Kaiso^Tg/+^* mice develop inflammation, possibly by altering p120^ctn^ localization and consequently function (Figure 8). Kaiso’s inhibition of the Notch pathway may hinder the ability of these mice to repair and regenerate the epithelium in response to inflammation, resulting in chronic inflammation that increases in severity over time, thus making the mice more susceptible to inflammation-induced tumorigenesis.

## Supporting Information

Figure S1 **Ectopic Kaiso expression in the intestine of** ***Kaiso^Tg/+^*** **mice.** **(A)** Line B *Kaiso^Tg/+^* mice display sporadic nuclear expression and strong cytoplasmic Kaiso expression in the epithelial cells of the villi but lack Kaiso expression in the crypts, compared to Non-Tg mice. **(B)** In the colon, Non-Tg mice display low nuclear Kaiso expression in the crypts, while Line A and B *Kaiso^Tg/+^* show strong nuclear Kaiso expression, with the apical epithelial cells displaying the most Kaiso expression. Line A colons show greater Kaiso expression than Line B colons.
(TIF)

Figure S2 **Ectopic Kaiso expression in the small intestine of multiple Kaiso transgenic lines induces inflammatory cell infiltration.** *Kaiso^Tg^* ^/+^ mice display strong nuclear Kaiso expression in the villi and crypt cells, however Non-Tg mice display weak Kaiso expression with most Kaiso localizing to the cytoplasm. Line E and F (generation 3) show strong Kaiso expression from the base of the crypts to the top of the villi. Interestingly, in all three *Kaiso^Tg^* ^/+^ lines analysed, ectopic Kaiso expression also appears to induce villi fusion (black arrows). Histological analysis showed increased neutrophil infiltration into the villi of Lines A, E and F *Kaiso^Tg^* ^/+^ mice (yellow demarcated area). An MPO assay of Line A, E and F ileums show increased MPO activity when compared to age-matched Non-Tg. Immunofluorescence revealed nuclear p120^ctn^ in both Line E and F in the villi.
(TIF)

Figure S3 **Line A** ***Kaiso^Tg/+^*** **mice display increased numbers of differentiated cells in the colon.** *Kaiso^Tg/+^* mice display a significant increase in Goblet (PAS stain), and enteroendocrine cells (synaptophysin) in the large intestine (colon) compared to their Non-Tg littermates.
(TIF)

Figure S4 **Kaiso expression is increased in DSS-treated murine colon tissues.** Preliminary analysis of DSS-induced murine colitis model intestinal tissues revealed increased Kaiso nuclear expression in DSS-treated colon tissues whereas non-treated mice show low cytoplasmic Kaiso expression.
(TIF)
