# Supplementary figures and images for: The POZ-ZF Transcription Factor Kaiso (ZBTB33) Induces Inflammation and Progenitor Cell Differentiation in the Murine Intestine
Source: PLoS One. 2013 Sep 5;8(9):e74160. doi: 10.1371/journal.pone.0074160 (PMC3764064; doi:10.1371/journal.pone.0074160)

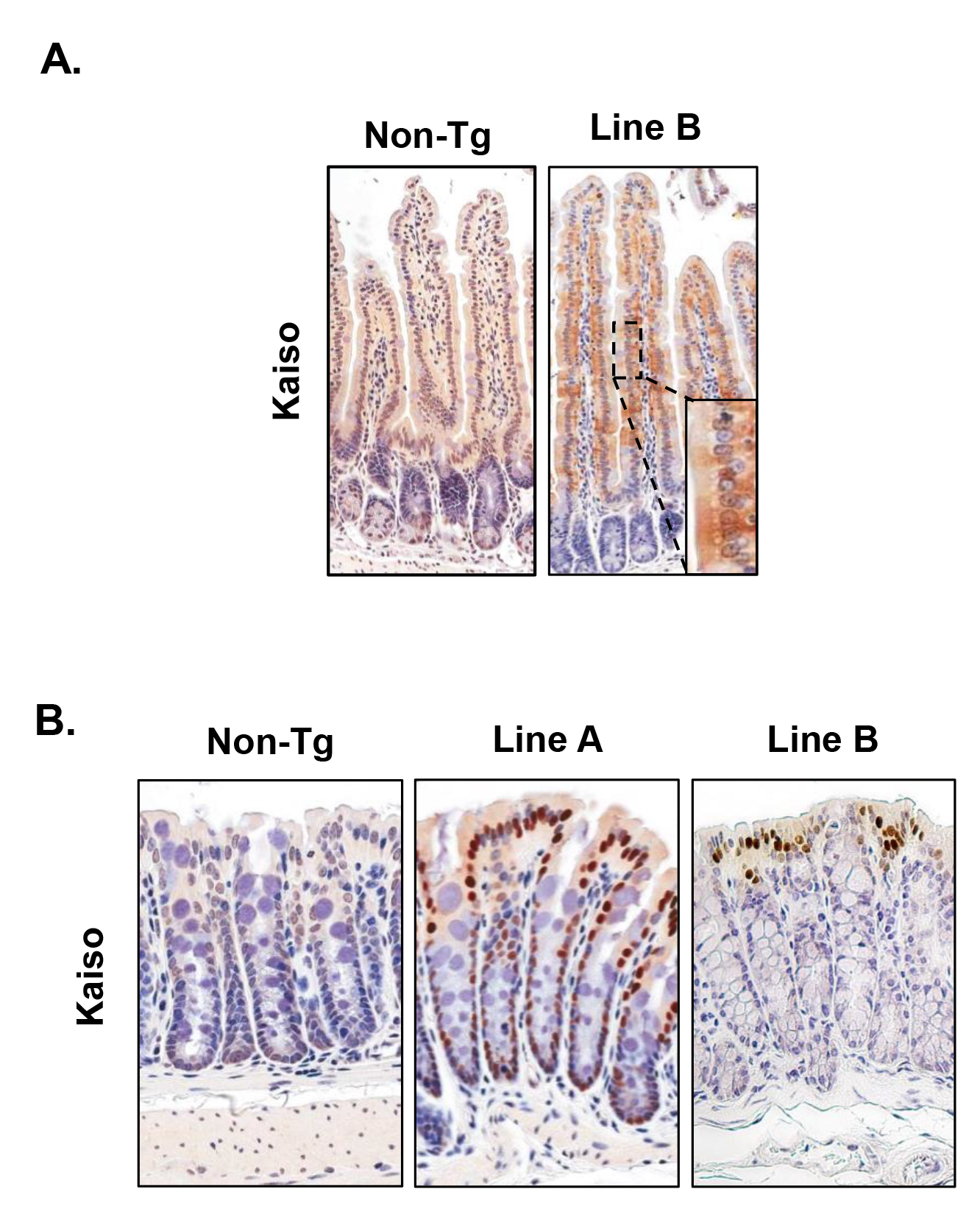

Supplement: Figure S1 — Ectopic Kaiso expression in the intestine of KaisoTg/+ mice. (A) Line B KaisoTg/+ mice display sporadic nuclear expression and strong cytoplasmic Kaiso expression in the epithelial cells of the villi but lack Kaiso expression in the crypts, compared to Non-Tg mice. (B) In the colon, Non-Tg mice display low nuclear Kaiso expression in the crypts, while Line A and B KaisoTg/+ show strong nuclear Kaiso expression, with the apical epithelial cells displaying the most Kaiso expression. Line A colons show greater Kaiso expression than Line B colons. (TIF) [file pone.0074160.s001.tif]

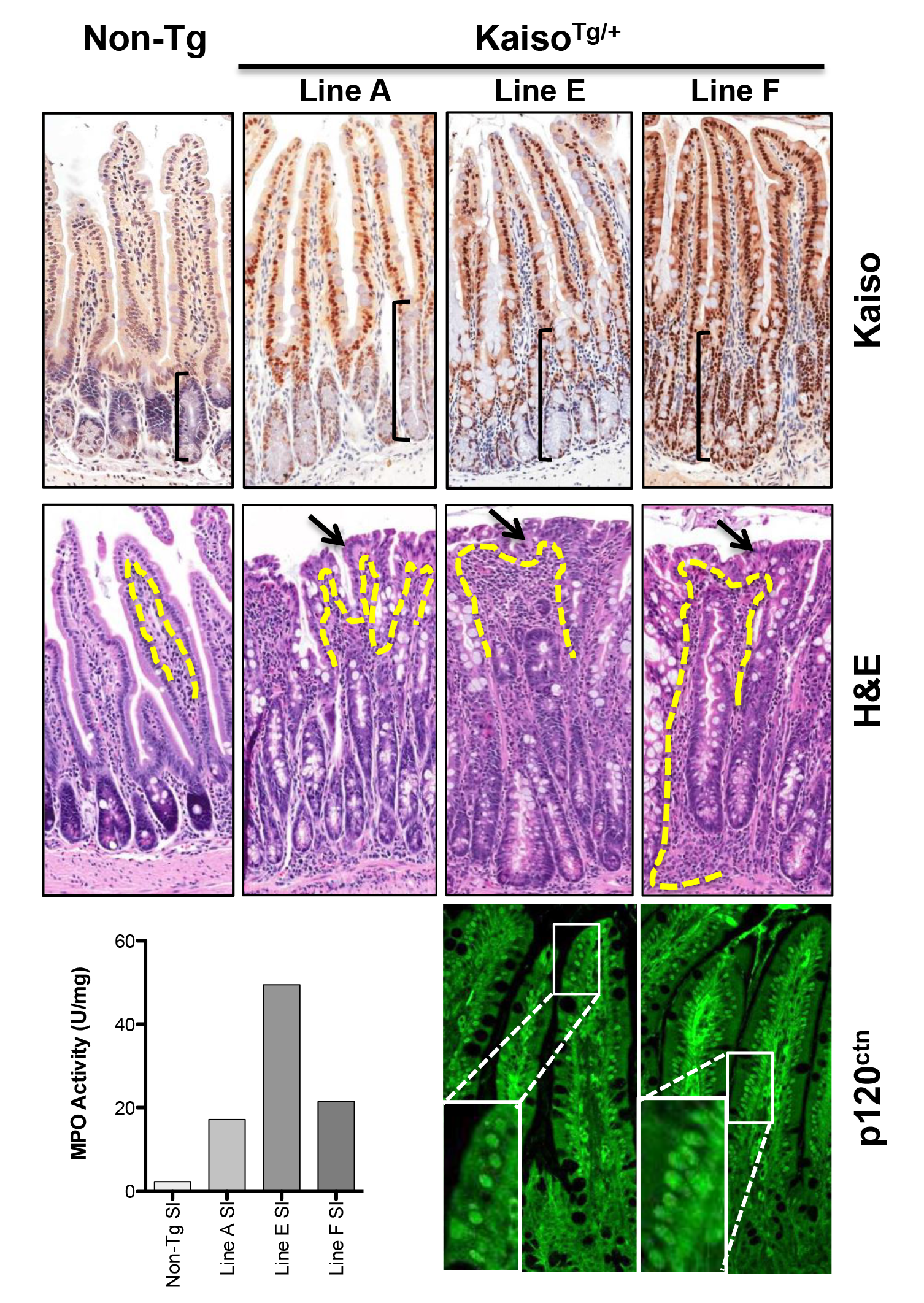

Supplement: Figure S2 — Ectopic Kaiso expression in the small intestine of multiple Kaiso transgenic lines induces inflammatory cell infiltration. KaisoTg /+ mice display strong nuclear Kaiso expression in the villi and crypt cells, however Non-Tg mice display weak Kaiso expression with most Kaiso localizing to the cytoplasm. Line E and F (generation 3) show strong Kaiso expression from the base of the crypts to the top of the villi. Interestingly, in all three KaisoTg /+ lines analysed, ectopic Kaiso expression also appears to induce villi fusion (black arrows). Histological analysis showed increased neutrophil infiltration into the villi of Lines A, E and F KaisoTg /+ mice (yellow demarcated area). An MPO assay of Line A, E and F ileums show increased MPO activity when compared to age-matched Non-Tg. Immunofluorescence revealed nuclear p120ctn in both Line E and F in the villi. (TIF) [file pone.0074160.s002.tif]

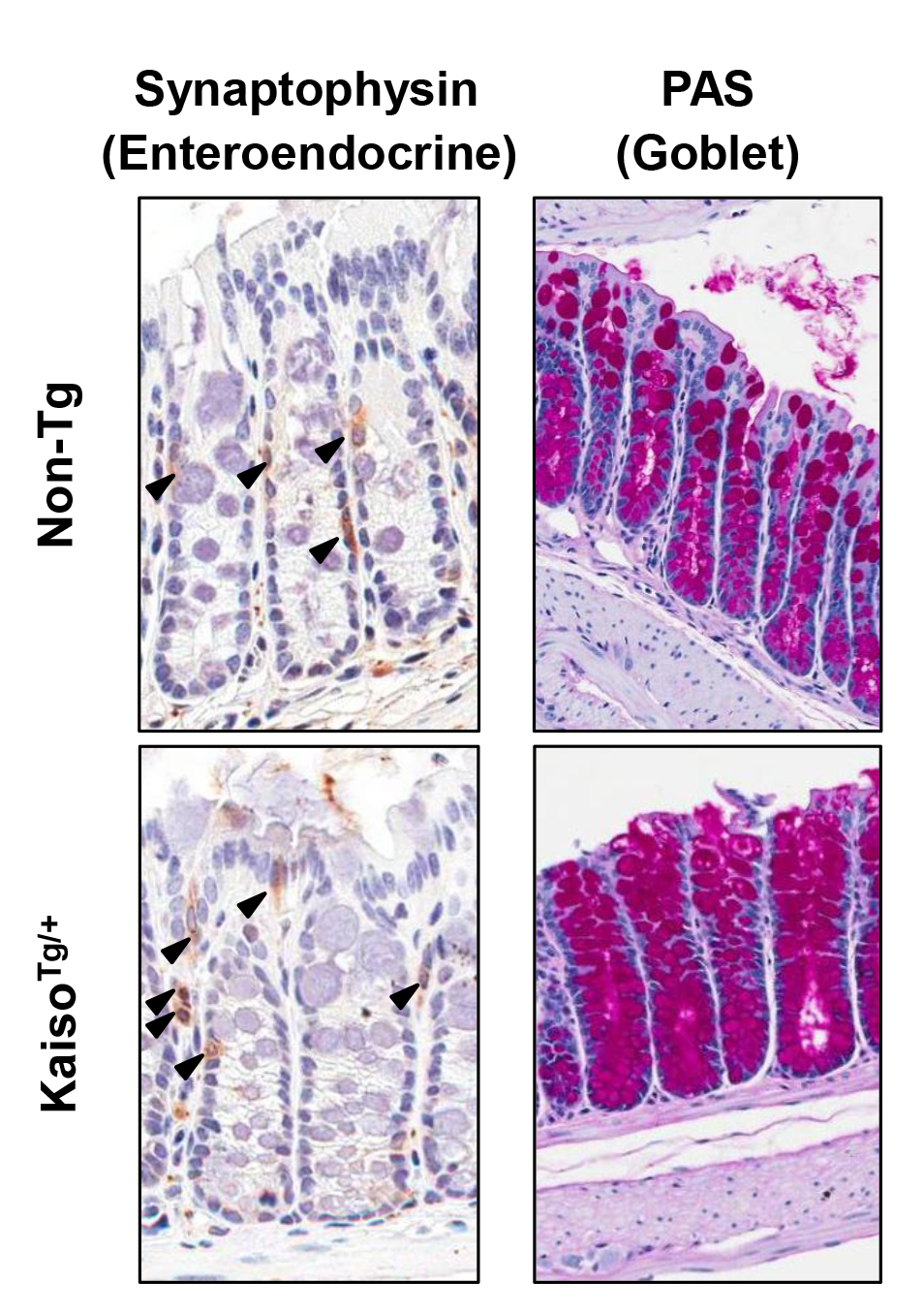

Supplement: Figure S3 — Line A KaisoTg/+ mice display increased numbers of differentiated cells in the colon. KaisoTg/+ mice display a significant increase in Goblet (PAS stain), and enteroendocrine cells (synaptophysin) in the large intestine (colon) compared to their Non-Tg littermates. (TIF) [file pone.0074160.s003.tif]

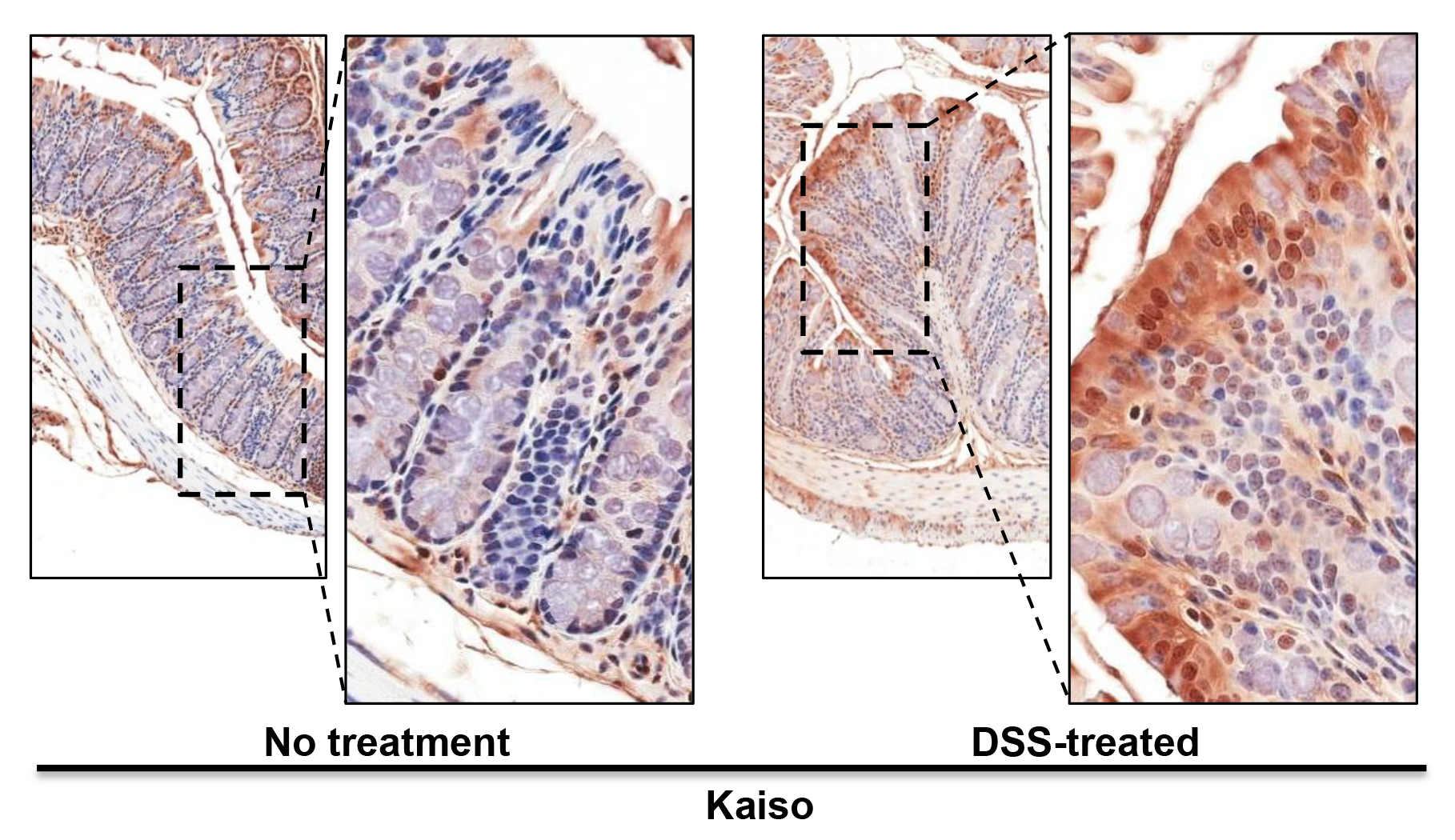

Supplement: Figure S4 — Kaiso expression is increased in DSS-treated murine colon tissues. Preliminary analysis of DSS-induced murine colitis model intestinal tissues revealed increased Kaiso nuclear expression in DSS-treated colon tissues whereas non-treated mice show low cytoplasmic Kaiso expression. (TIF) [file pone.0074160.s004.tif]
